# Amino Acid and Fatty Acid Metabolism Disorders Trigger Oxidative Stress and Inflammatory Response in Excessive Dietary Valine-Induced NAFLD of Laying Hens

**DOI:** 10.3389/fnut.2022.849767

**Published:** 2022-04-12

**Authors:** Huafeng Jian, Qianqian Xu, Xiaoming Wang, Yating Liu, Sasa Miao, Yan Li, Tianming Mou, Xinyang Dong, Xiaoting Zou

**Affiliations:** ^1^Institute of Feed Science, College of Animal Sciences, Zhejiang University, Hangzhou, China; ^2^The National Engineering Laboratory for Feed Safety and Pollution Prevention and Controlling, National Development and Reform Commission, Zhejiang University, Hangzhou, China; ^3^Key Laboratory of Molecular Animal Nutrition, Ministry of Education, Zhejiang University, Hangzhou, China; ^4^Key Laboratory of Animal Nutrition and Feed Science, Ministry of Agriculture and Rural Affairs, Zhejiang University, Hangzhou, China; ^5^Key Laboratory of Animal Feed and Nutrition of Zhejiang Province, Zhejiang University, Hangzhou, China

**Keywords:** amino acid imbalance, fatty acid metabolism, TORC1, autophagy, NAFLD, laying hens

## Abstract

Non-alcoholic fatty liver disease (NAFLD) is a chronic and metabolic liver disease and commonly occurs in humans with obesity and type 2 diabetes mellitus (T2DM); such a condition also exists in animals such as rodents and laying hens. Since the pathogenesis of fatty liver hemorrhagic syndrome (FLHS) of laying hens is similar to human NAFLD, hen's FLHS is commonly selected as a study model of NAFLD. Altered circulating amino acids, particularly elevated branched-chain amino acids (BCAAs) and aromatic amino acids (AAAs), are consistently reported in patients with NAFLD and T2DM. How long-term dietary individual BCAA, such as valine, impacts amino acid and fatty acid metabolism remains unknown. In this study, we demonstrated that when laying hens are fed with dietary valine at different levels (59, 0.64, 0.69, 0.74, and 0.79%) in a feeding trial that lasted for 8 weeks, long-term exposure to excessive valine diets at 0.74 and 0.79% levels could induce amino acid imbalance, impair amino acid metabolism, increase fatty acid synthesis, and inhibit fatty acid utilization. Long-term intake of excessive dietary valine could result in impaired amino acid metabolism *via* inhibiting C/EBP-β/asparagine synthetase (Asns). This process is mediated by downregulating the general control nonderepressible-eukaryotic initiation factor 2α- activating transcription factor (GCN2-eIF2α-ATF4) pathway and elevating corresponding circulating BCAAs and AAAs levels, which could ultimately result in amino acid imbalance. High levels of dietary valine stimulated lipid deposition by suppressing the GCN2-eIF2α-ATF4-fibroblast growth factor-19 (FGF19)-target of rapamycin complex 1 (TORC1) signaling pathway to promote fatty acid synthesis, repress fatty acid utilization, and eventually accelerate the development of NAFLD. The Spearman correlation analysis revealed that circulating amino acid imbalance is significantly associated with fatty acid metabolism disorder and enhanced oxidative stress. The inhibition of the GCN2-TORC1 pathway induced autophagy suppression to trigger liver oxidative stress and inflammatory response. In conclusion, our results revealed the adverse metabolic response to excessive dietary valine mediated by amino acid and fatty acid metabolism disorders. This study also suggested reducing dietary valine as a novel approach to preventing and treating NAFLD in humans and FLHS in laying hens.

## Introduction

Non-alcoholic fatty liver disease (NAFLD) is one of the most common metabolic and chronic liver. Its prevalence and incidence are increasing and affect one-fourth of the population worldwide (1). The characteristics of NAFLD include a series of liver pathologies ranging from simple hepatic steatosis, non-alcoholic steatohepatitis (NASH), fibrosis, and cirrhosis (2). Hepatic lipid deposition [including elevated free fatty acids (FFAs) and triglycerides (TGs)], steatosis, insulin resistance, oxidative stress, inflammatory response, endoplasmic reticulum (ER) stress, and autophagy inhibition are the main characters of NAFLD. The mechanisms of NAFLD which favor the development of hepatic steatosis and transition to NASH, cirrhosis, and hepatocellular carcinoma (HCC) are still not fully understood (2–4). The original model of NASH pathogenesis was the “two-hit” hypothesis which dictated that hepatic lipid accumulation provides a “first hit” that sensitized the liver to a “second hit” of oxidative stress due to increased lipotoxicity and subsequent necro-inflammation that precipitated NASH (5, 6). The chronic metabolic challenge of hepatocytes with oxidative and ER stress caused by lipotoxicity and necro-inflammation leads to cellular damage or cell death and disease progression resulting in immune cell infiltration, fibrogenesis, and activation of hepatic progenitor cells (7–9). Lipotoxicity induced by excessive lipids such as FFAs and TGs involves ER stress, oxidative stress, mitochondrial dysfunction, impaired autophagy, and inflammation (10). Lipid-induced autophagy, for instance, lipid overload facilitates autophagy of lipid droplets (LDs, lipophagy) (11). To date, there is an increasing burden of NAFLD due to the pathogenesis complexity of NAFLD and the lack of available therapies.

In addition to altered lipid metabolism in the progression of NAFLD, several reports have indicated that amino acid metabolism imbalance has been associated with an increased risk and disease severity of NAFLD (12–15). For instance, elevated serum or plasma concentrations of BCAAs, such as leucine, isoleucine, valine, and aromatic amino acids (AAAs, e.g., tyrosine, phenylalanine, and tryptophan), are found in the T2DM and NAFLD in humans and are associated with increased severity of liver diseases (16–19). Apart from increased BCAAs and AAAs, glutamine, glutamate, alanine, and aspartate are positively associated with increased hepatic insulin resistance, whereas decreased glycine and serine are found in humans with NAFLD (20–23). The BCAAs leucine, isoleucine, and valine have assumed particular prominence, both because of their role in affecting mammalian target of rapamycin (mTOR)-key pathways linking nutrition with health and aging (24) and also because their circulating levels are positively associated with obesity, insulin resistance, and metabolic dysfunction in rodents and humans (16, 25, 26). As an important nutrition sensor, mTOR consists of mTORC1 and mTORC2, and the mTORC1 can be activated by branched-chain amino acids (BCAAs) which subsequently inhibit autophagy (27). During amino acid sufficiency, mTORC1 is activated and resulting in dissociation of the UNC-like autophagy-activating kinase 1 (ULK1) active complex and inhibition of autophagy (11, 27). In addition to mTOR, eukaryotic initiation factor 2α (eIF2α) kinase general control nonderepressible (GCN2) can also function as an amino acid sensor to promote transcription of autophagy genes (11). During amino acid starvation, GCN2 suppressed intestinal inflammation by inhibiting inflammasome activation and triggering autophagy in mice, and eventually decreased the production of pro-inflammatory cytokines interleukin (IL)-1β and IL-17 (28). GCN2 might detect a paucity of one or more essential amino acids to inhibit mTORC1, resulting in autophagy induction (29).

In poultry, specifically the laying hens, fatty liver hemorrhagic syndrome (FLHS) resulting from NAFLD has been a significant cause of death in commercial layers (30). The pathogenesis of FLHS is similar to NAFLD in humans, which is characterized by increased hepatic TG content accompanied by liver hemorrhage and large amounts of lipid accumulation in the abdominal cavity, which usually causes considerable mortality of laying hens during the peak laying period owing to liver rupture resulting in internal bleeding (31). Our recent reports demonstrated that diet-supplemented valine has significantly altered the serum-free amino acid profile of laying hens. Additionally, long-term exposure to high levels of dietary valine at 0.74 and 0.79% has accelerated the development of NAFLD of laying hens by promoting lipogenesis and inhibiting fatty acid oxidation mediated by GCN2-eIF2α-activating transcription factor 4 (ATF4) (32, 33). NAFLD induced by excessive dietary valine resulted in strengthening oxidative stress, ER stress, and inflammatory response (32, 33). Nevertheless, a comprehensive investigation of the roles of dietary valine in the change of amino acid profile and fatty acid metabolism in NAFLD has not been fully pursued.

In the current study, we sought to determine whether and how dietary valine manipulation impacts serum amino acid balance, amino acid metabolism, fatty acid metabolism, and the relationship between dietary valine and amino acid and fatty acid metabolism. We further analyzed whether strengthening oxidative stress and inflammatory response was mediated by the mTORC1-autophagy pathway. To test this hypothesis, we used adult laying hens as a model fed with different levels of dietary valine for 8 weeks and examined the impacts of dietary valine supplementation on serum-free amino acid profile and fatty acid metabolism. We also examined whether enhanced oxidative stress and inflammatory response are mediated by a mTORC1-autophagy pathway, which is a downstream signaling pathway of GCN2-eIF2α-ATF4.

## Materials and Methods

### Diets, Birds, and Management

Corn and soybean meals were selected as major ingredients to make up a corn-soybean-type basal diet and prepared according to NRC (1994) and China's “Chicken Feeding Standard (NY/T33-2004)” (34, 35). Synthetic L-Val (98% purity, Specom Biochemical Co. Ltd, Zhangjiagang, China) was supplemented to the basal diet in 0, 0.0508, 0.1016, 0.1523, and 0.2031% increments, resulting in experimental diets containing 0.59, 0.64, 0.69, 0.74, and 0.79% valine, respectively (Table 1). In addition, the ratio of other amino acids of the diet was corrected to be consistent with each group by dietary protein.

**Table 1:** Composition and nutrient levels of the basal diet (air-dry basis).

**Ingredients**	**Dietary valine levels (%)** ^**a**^				
	0.59	0.64	0.69	0.74	0.79
Corn	66.6	66.6	66.6	66.6	66.6
Soybean meal	10.5	10.65	11.2	11.55	11.8
Wheat bran	2.9	2.9	2.91	2.92	2.92
Peanut meal	8.7	8.5	7.9	7.5	7.2
Limestone	9.3	9.3	9.3	9.3	9.3
Soybean oil	0.3	0.3	0.3	0.3	0.3
DL-Methionine (98%)	0.16	0.16	0.15	0.15	0.15
Lysine (78%)	0.11	0.11	0.11	0.1	0.1
Valine (98%)	0	0.0508	0.1016	0.1523	0.2031
CaHPO4	0.6	0.6	0.6	0.6	0.6
Salt	0.36	0.36	0.36	0.36	0.36
Choline chloride, 60%	0.2	0.2	0.2	0.2	0.2
Mineral and vitamin premix^b^	0.27	0.27	0.27	0.27	0.27
**Calculated nutritional level, %**					
Crude protein (CP) (kcal/g)	14.7 (58.8)	14.7 (58.8)	14.7 (58.8)	14.7 (58.8)	14.7 (58.8)
ME, MJ/Kg (kcal/g)	2.68 (2.70)	2.68 (2.70)	2.68 (2.70)	2.68 (2.70)	2.68 (2.70)
Weight Fat/Carbohydrate	37.04	37.04	37.04	37.04	37.04
Fat, kcal/g	9	9	9	9	9
Carbohydrate, kcal/g	10.8	10.8	10.8	10.8	10.8
**Analyzed nutritional level, %**					
Crude protein (CP)	14.65	14.72	14.75	14.74	14.78
Calcium (calculated)	3.58	3.58	3.59	3.59	3.59
Total phosphorus	0.46	0.46	0.46	0.46	0.46
Methionine	0.36	0.36	0.36	0.37	0.37
Lysine	0.66	0.66	0.66	0.67	0.67
Threonine	0.48	0.48	0.48	0.49	0.49
Tryptophan	0.14	0.14	0.14	0.14	0.14
Arginine	1.05	1.04	1.03	1.03	1.02
Leucine	0.82	0.82	0.82	0.82	0.82
Isoleucine	0.65	0.65	0.65	0.65	0.65
Valine	0.59	0.64	0.69	0.74	0.79

a *Analyzed value of pooled experimental diets 0.59, 0.64, 0.69, 0.74, and 0.79% valine*.

b *The premix provided following per kilogram of diet: vitamin A, 7,500 IU; vitamin D3, 2500 IU; vitamin E, 49.5 mg; vitamin K3, 2.5 mg; vitamin B1, 1.5 mg; vitamin B2, 4 mg; vitamin B6, 2 mg; vitamin B12, 0.02 mg; niacin, 30 mg; folic acid, 1.1 mg; pantothenic acid, 10 mg; biotin,0.16 mg; chloride choline, 400 mg; Sodium chloride, 2,500 mg; Fe, 80 mg; Cu, 20 mg; Mn, 60 mg; Zn, 80 mg; I, 0.8 mg*.

A total of 960 healthy 33-weeks-old Fengda No. 1 laying hens with similar body weight and laying rate were randomly allocated into 5 experimental groups, and each group included 6 replicates of 32 laying hens (8 birds/cage). This study lasted 9 weeks, including a 1-week acclimation period and an 8-week experimental period. All hens were housed in an environmentally controlled room where the temperature was maintained at approximately 23°C. The hens were exposed to a 16 h photoperiod throughout the experiment by the use of artificial lighting. Hens were supplied with water and fed a complete feeding mixture twice daily. All animal works in this experiment were conducted by following the Chinese Guidelines for Animal Welfare and approved by the Zhejiang University Institutional Animal Care and Use Committee (No. ZJU2013105002) (Hangzhou, China).

### Sample Collection and Processing

At the end of the 8-week experiment, 2 hens were randomly selected from each replication (12 hens in each group; a total of 60 hens) and fasted for 12 h. A blood sample (5 ml; bird-1) was collected from the vein under the wing. After centrifugation at 3,000 × *g* for 10 min, serum was separated. After blood sampling, hens were euthanized with pentobarbital sodium and sacrificed. The liver was collected for the determination of liver lipid metabolism parameters and mRNA expression.

### Liver Parameter Measurements

One gram liver was homogenized in 9 ml of 0.9% (w/v) sterile normal saline on ice and centrifuged at 3,500 × *g* at 4°C for 15 min. Total protein in the tissue supernatant was measured with a BCA protein assay kit (Nanjing Jiancheng Bioengineering Institute, Nanjing, China) according to the manufacturer's protocol and stored at −80°C storage. The concentrations of non-esterified fatty acid (NEFA), high-density lipoprotein cholesterol (HDL), and low-density lipoprotein cholesterol (LDL) were determined using commercial kits (Nanjing Jiancheng Bioengineering Institute, Nanjing, China). All assays were performed according to the manufacturer's instructions.

### ELISA Determination

The concentrations of fatty acid synthase (FASN), acetyl-CoA carboxylase (ACC), ATP citrate lyase (ACLY), and very-low-density lipoprotein cholesterol (VLDL) in the liver were determined using chicken-specific ELISA quantitation kits (Nanjing Jiancheng Bioengineering Institute, Nanjing, China). All assays were performed according to the manufacturer's instructions.

### Total RNA Isolation and Relative Quantitative RT-PCR

The liver mRNA expression level was determined using real time-PCR. Total RNA was extracted using TRIzol reagent (Takara code: 9109, Shiga, Japan). RNA quality and quantity were determined using a NanoDrop 2000 spectrophotometer (Thermo Fisher Scientific, Massachusetts, USA). cDNA was synthesized with a HiScriptIIqRT SuperMix Reverse Transcriptase (Vazyme Biotechnology, Nanjing, Jiangsu, China). Real-time PCR was conducted with a SYBR Premix PCR kit (Vazyme Biotechnology, Nanjing, Jiangsu, China) *via* the CFX96TM Real-Time System (Bio-Rad, Hercules, CA, USA). Table 2 shows gene-specific primers for RT-PCR. The reference gene β-actin was used as an internal control. Each sample was run in triplicate, and the 2^−Δ*ΔCt*^ method was employed for evaluating the relative mRNA expression level of the target gene.

**Table 2:** Primers used for quantitative real-time PCR.

**Gene**	**Primer**	**Primer sequence (5'-3')**	**GenBank number**
β-Actin	Forward	TCCCTGGAGAAGAGCTATGAA	NM_205518.1
	Reverse	CAGGACTCCATACCCAAGAAAG	
FABP1	Forward	GCAGAATGGGAATAAGTT	NM_204192.4
	Reverse	TTGTATGGGTGATGGTGT	
FATP1	Forward	TACAATGTGCTCCAGAAGGG	NM_001039602.2
	Reverse	GTCTGGTTGAGGATGTGACTC	
CD36	Forward	ACTGCGCTTCTTCTCCTCTGA	NM_001030731.1
	Reverse	TCACGGTCTTACTGGTCTGGTAAA	
apoA1	Forward	GTGACCCTCGCTGTGCTCTT	NM_205525.5
	Reverse	CACTCAGCGTGTCCAGGTTGT	
apoB	Forward	ATGTTCAAAAGATGCGGCCC	NM_001044633.2
	Reverse	GCATGGCTCTTCTCTCACTG	
C/EBP-β	Forward	ACGAGGCGGACTGTTTGG	NM_205253.3
	Reverse	GCTGCTGGGATGCTGCTAA	
Asns	Forward	ACCACTCCATGCTGCTTGTG	NM_001030977.2
	Reverse	ATCCAAGCCCCCTGACAAAA	
LC3I	Forward	ATGGCAGAGGTGTACAGGGACTAC	XM_419549.6
	Reverse	GGGTGAGTGAGCAGCATCCAAAC	
LC3II	Forward	AGTGAAGTGTAGCAGGATGA	NM_001031461.1
	Reverse	AAGCCTTGTGAACGAGAT	
ULK1	Forward	GAGCAAGAGCACACCGACATCC	XM_015275648.2
	Reverse	TTTCAGGGCAGCAATCTCCATCAC	
Atg5	Forward	GCCTTCAGTGGGGTTTCAGTTCC	XM_015284517.2
	Reverse	TATGCGTCCAAACCACACATCTCG	
Atg7	Forward	TCAGATTCAAGCACTTCAGA	NM_001030592.1
	Reverse	GAGGAGATACAACCACAGAG	
FGF19	Forward	CCCGCTGTCTCACTTCTTACCCA	NM_204674.3
	Reverse	GGATCCATGCTGTCCGTTTCG	
UCP3	Forward	ACTCTGTGAAGCAGCTCTACACC	NM_204107.1
	Reverse	ATGTACCGCGTCTTCACCACATC	
TORC1	Forward	GGACTCTTCCCTGCTGGCTAA	XM_417614.5
	Reverse	TACGGGTGCCCTGGTTCTG	

### Spearman Correlation Analysis

The Spearman correlation analysis was analyzed on the free online platform of LC-Bio Cloud Platform (https://www.omicstudio.cn/). In brief, Spearman correlation analysis was conducted to examine the association between dietary valine levels and serum-free amino acids, serum-free amino acids and lipid metabolism parameters, serum-free amino acids, and anti-oxidase parameters using LC-Bio Cloud Platform (https://www.omicstudio.cn/). The sample size and sample individual of the Spearman correlation analysis in the current research was a one-to-one correspondence between serum free amino acids and lipid metabolism parameters and anti-oxidase parameters (*n* = 4). The results of the Spearman correlation analysis were shown in a heatmap and ^*^ represents *P* < 0.05 and ^**^ represents *P* < 0.01.

### Statistical Analysis

The Gaussian distribution of data was analyzed by the Kolmogorov–Smirnov test using SPSS 20 (Chicago, IL, USA). The variance of the data was analyzed by the homogeneity of variance test (SPSS 20). Statistical analysis was performed with one-way ANOVA followed by LSD's multiple comparison tests with SPSS 20. The linear or quadratic verification was performed by SPSS 20. Data presented in the article are shown as means ± SEM and are considered significant at *P* < 0.05.

## Results

### NAFLD Driven by Excessive Dietary Valine-Altered Serum Free Amino Acid Profile and Liver Amino Acid Metabolism Mediated by GCN2-EIF2α-ATF4

**Figure 1:**
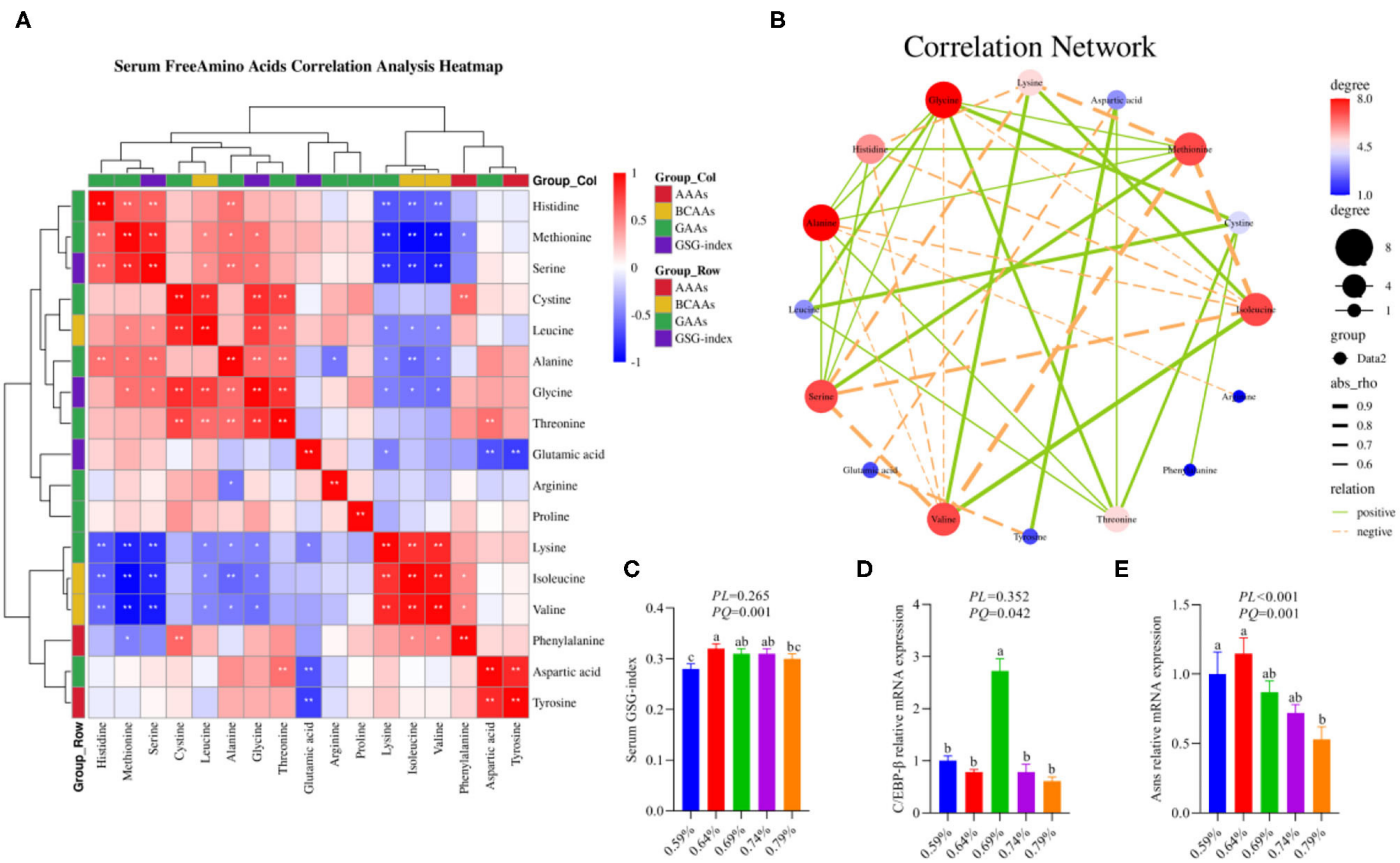
Dietary valine changed serum free amino acid profile and liver amino acid metabolism (*n* = 6–8). **(A)** Serum-free amino acids correlation analysis heatmap. **(B)** Correlation network. **(C)** Serum GSG-index. **(D)** C/EBP-β relative mRNA expression level. **(E)** Asns relative mRNA expression level. *P < 0.05 and **P < 0.01. ^a−*c*^Means with different superscripts within a column differ significantly (*P* < 0.05). GSG index, Glutamate–serine–glycine (GSG) index [glutamate/(serine1glycine)]; AAAs, aromatic amino acids; BCAAs, branched-chain amino acids; GAAs, glycogenous amino acids.

Our previous results demonstrated that dietary valine treatment significantly elevated serum-free Isoleucine (Ile), Lysine (Lys), Phenylalanine (Phe), Valine (Val), and Tyrosine (Tyr) with increasing of dietary valine levels, whereas decreased serum free Arginine (Arg), Histidine (His), Methionine (Met), Threonine (Thr), Alanine (Ala), Aspartic acid (Asp), Glutamic acid (Glu), Glycine (Gly), Leucine (Leu), and Serine (Ser) (32). In addition, there is no difference was observed in serum-free Proline (Pro) and Cystine (Cys) among all treatments (32). Elevated circulating levels of BCAAs and AAAs have been reported in patients with NAFLD (16–19). Based on significantly changed serum free amino acid profile, we further sought to determine the relationship of dietary valine levels and altered serum free amino acids by Spearman correlation analysis. The Spearman correlation analysis heatmap revealed that serum free His was positively associated with Ala, Ser, and Met, while was negatively associated with Val, Ile, and Lys (*P* < 0.01); Met was positively associated with Gly, Ala, Leu, Ser, and His, but was negatively associated with Phe, Val, Ile, and Lys (*P* < 0.05); Ser was positively associated with Gly, Ala, Leu, Met, and His, whereas was negatively associated with Val, Ile, and Lys (*P* < 0.05); Cys was positively associated with Phe, Thr, Gly, and Leu (*P* < 0.01); Leu was positively associated with Thr, Gly, Cys, Ser, and Met, yet was negatively associated with Val, Ile, and Lys (*P* < 0.05); Ala was positively associated with Thr, Gly, Ser, Met, and His, whereas was negatively associated with Val, Ile, Lys, and Arg (*P* < 0.05); Gly was positively associated with Thr, Ala, Leu, Cys, Ser, and Met, whereas was negatively associated with Val, Ile, and Lys (*P* < 0.05); Thr was positively associated with Asp, Gly, Ala, Leu, and Cys (*P* < 0.01); Glu was negatively associated with Tyr, Asp, and Lys (*P* < 0.05); Arg was negatively associated with Ala (*P* < 0.05); Lys was positively associated with Val and Ile, but was negatively associated with Glu, Gly, Ala, Leu, Ser, Met, and His (*P* < 0.05); Ile was positively associated with Phe, Val, and Lys, yet was negatively associated with Gly, Ala, Leu, Ser, Met, and His (*P* < 0.05); Val was positively associated with Phe, Ile, and Lys, whereas was negatively associated with Gly, Ala, Leu, Ser, Met, and His (*P* < 0.05); Phe was positively associated with Val, Ile, and Cys, but was negatively associated with Met (*P* < 0.05); Asp was positively associated with Tyr and Thr, yet was negatively associated with Glu (*P* < 0.01); Tyr was positively associated with Asp, whereas was negatively associated with Glu (*P* < 0.01) (Figure 1A). The correlation network further revealed dietary valine treatment dramatically influencing the components of serum-free Gly, Met, Ile, Ala, Ser, Val, His, Lys, and Thr (Figure 1B). Taken together, the above results may indicate that NAFLD induced by excessive dietary valine changed amino acid profile by elevating BCAAs and AAAs, in particular, Ile, Val, Phe, and Tyr, and reducing Gly, Met, Ala, Ser, His, and Thr, and eventually lead to amino acid imbalance.

As shown in the correlation analysis heatmap and network, dietary valine supplementation significantly correlated with elevated concentrations of BCAAs (e.g., Ile and Val) and AAAs such as Phe and Tyr (Figure 1B), which has been confirmed that was associated with the development and progression of NAFLD (16–19). The glutamate–serine–glycine (GSG) index [glutamate/(serine1glycine)], a new marker of severity of liver disease independent of body mass index (12), showed a quadratic decrease with increasing dietary valine concentrations (*P* < 0.01) (Figure 1C). Compared with 0.59% dietary treatment (not supplement L-Val), the GSG-index of other four treatments were higher (*P* < 0.05) (Figure 1C). The changed serum-free amino acid profile may reflect altered amino acid metabolism in the NAFLD of laying hens. The mRNA expression levels of C/EBP-β and asparagine synthetase (Asns) revealed that dietary treatment significantly downregulated the mRNA expression levels of C/EBP-β and Asns, which is consistent with decreased serum free Arg, His, Met, Thr, Ala, Asp, Glu, Gly, Leu, and Ser, indicating NAFLD induced by excessive dietary valine dramatically impaired liver amino acid metabolism of laying hens (Figures 1D,E). In combination with the above results, these results clearly demonstrated that NAFLD induced by excessive dietary valine are both sufficient to alter serum free amino acid profile and damage liver amino acid metabolism, and eventually lead to amino acid imbalance.

### Excessive Dietary Valine Treatment Trigger NAFLD by Stimulating Liver Fatty Acid Synthesis

**Figure 2:**
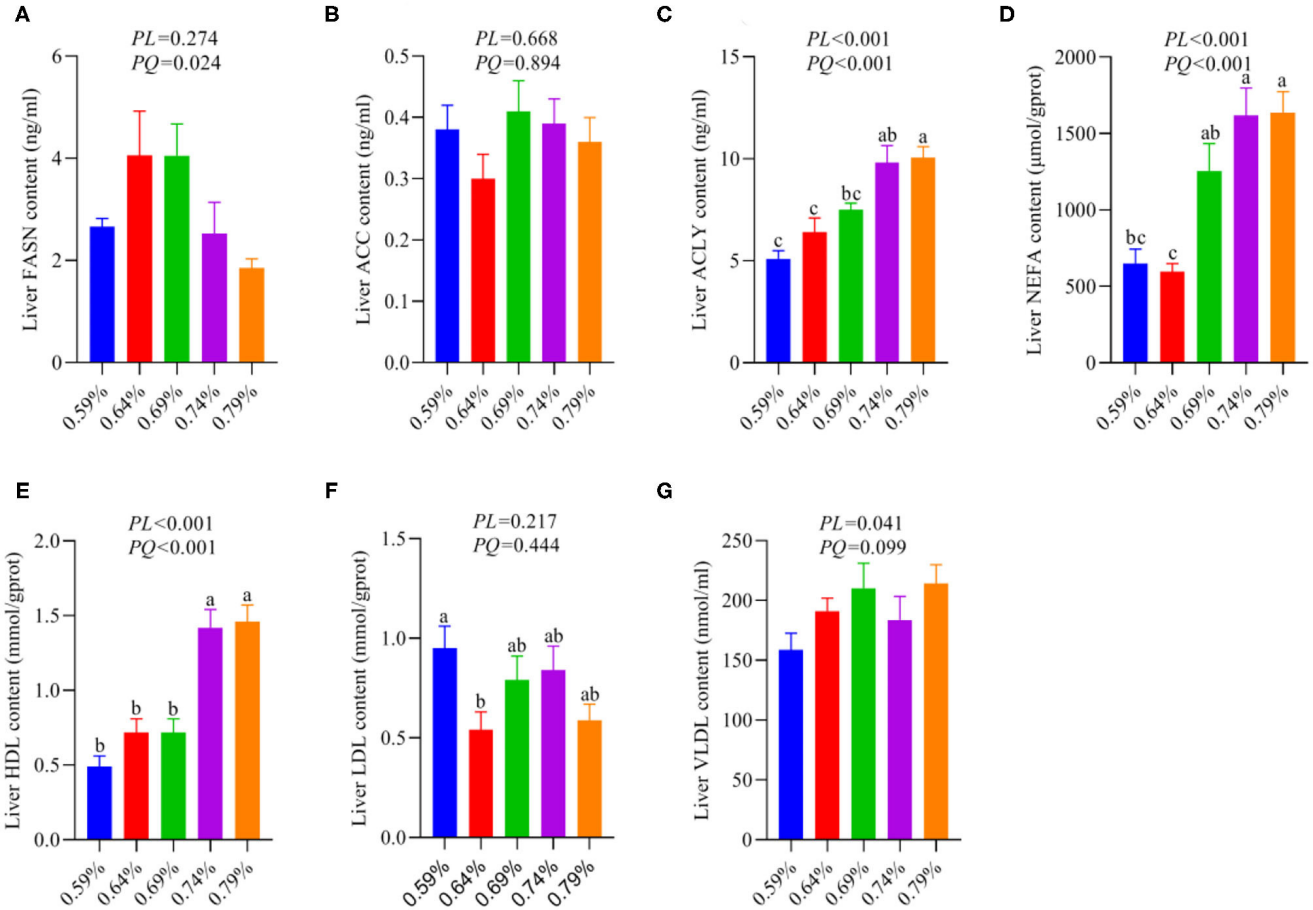
Effects of dietary valine levels on liver lipid parameters (*n* = 6–8). **(A)** FASN. **(B)** ACC. **(C)** ACLY. **(D)** NEFA. **(E)** HDL. **(F)** LDL. **(G)** VLDL. ^a−*c*^Means with different superscripts within a column differ significantly (*P* < 0.05).

As a substrate for the synthesis of TG, fatty acids can be esterified with glycerol sterol backbones, generating TGs or sterol esters (SEs), respectively, and then stored in LDs (36). Our recent report indicated dietary valine treatment dramatically increased the concentration of TG in the serum and liver with increasing dietary levels (33). We first investigated the effects of dietary valine on liver fatty acid synthesis, and we found that dietary valine supplementation dramatically improved the contents of liver ACLY, NEFA, HDL, and VLDL (*P* < 0.05) (Figures 2C,D,E,G), whereas the content of liver FASN showed an increase first and then decreased with the increase of dietary valine levels (*P* < 0.05) (Figure 2A). Dietary valine treatment did not affect the contents of liver ACC and LDL among all groups (*P* > 0.05) (Figures 2B,F). Consistent with our previous results that dietary valine significantly upregulated the mRNA expression levels of FASN and ACLY yet did not affect the mRNA expression level of ACC (33). Taken together with our previous results, we can conclude that excessive dietary valine treatment drives NAFLD by promoting fatty acid synthesis.

### Dietary Valine Treatment Promoted Lipid Deposition Drive NAFLD by Suppressing Liver Fatty Acid Utilization

**Figure 3:**
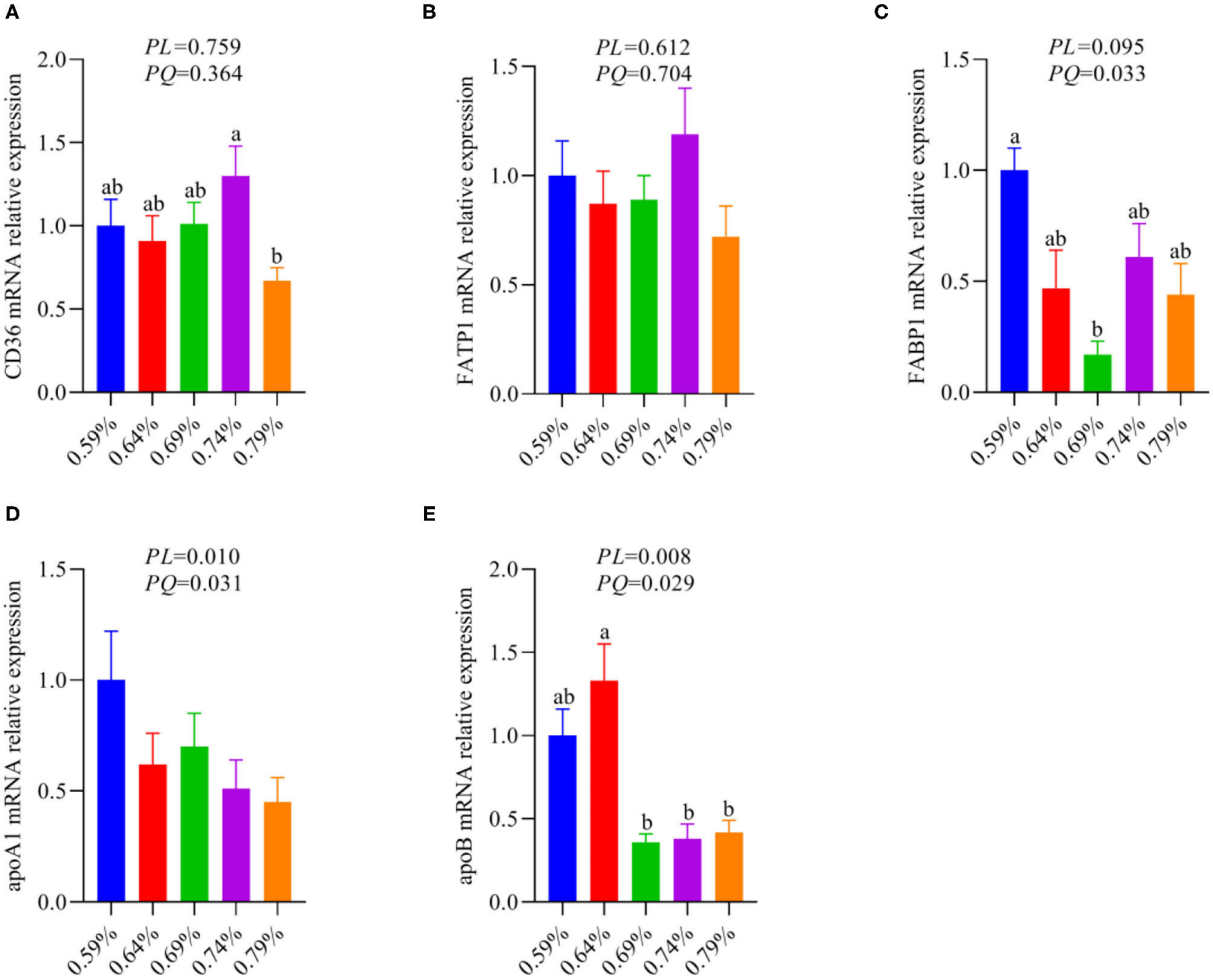
The relative mRNA expression levels of fatty acid metabolism associated genes in the liver of laying hens (*n* = 6–8). **(A)** CD36. **(B)** FATP1. **(C)** FABP1. **(D)** apoA1. **(E)** apoB. ^a, b^Means with different superscripts within a column differ significantly (*P* < 0.05).

The metabolism and utilization of hepatocyte fatty acid involving fatty acid synthesis, fatty acid uptake, fatty acid transportation, including import and export, and fatty acid oxidation. Our previous results suggested dietary valine supplementation significantly inhibited fatty acid β-oxidation *via* downregulating the mRNA expression levels of CPT1, ACOX1, and PPARα (33). Thus, we further investigated whether dietary valine treatment impact hepatocyte fatty acid metabolisms such as fatty acid uptake, fatty acid transportation, including import and export. As shown in Figure 3, dietary valine supplementation dramatically downregulated the mRNA expression levels of fatty acid export associated genes including apolipoproteinA1 (apoA1) and apoB (Figures 3D,E), whereas the mRNA expression level of fatty acid-binding protein (FABP1) showed first decreased and then increased in a quadratic manner (*P* < 0.05) (Figure 3C). However, dietary valine treatment did not affect the mRNA expression levels of fatty acid uptake and fatty acid transportation associated genes such as fatty acid translocase (CD36) and fatty acid transport protein 1 (FATP1) (*P* > 0.05) (Figures 3A,B). In combination with previous results, we can conclude that dietary valine treatment promoted lipid deposition trigger NAFLD *via* suppressing fatty acid export and β-oxidation associated genes mRNA expression levels.

### Elevated Serum Free Amino Acid Val, Ile, and Lys Are Correlated With Serum TG, Liver NEFA, TG, HDL, and ACLY

**Figure 4:**
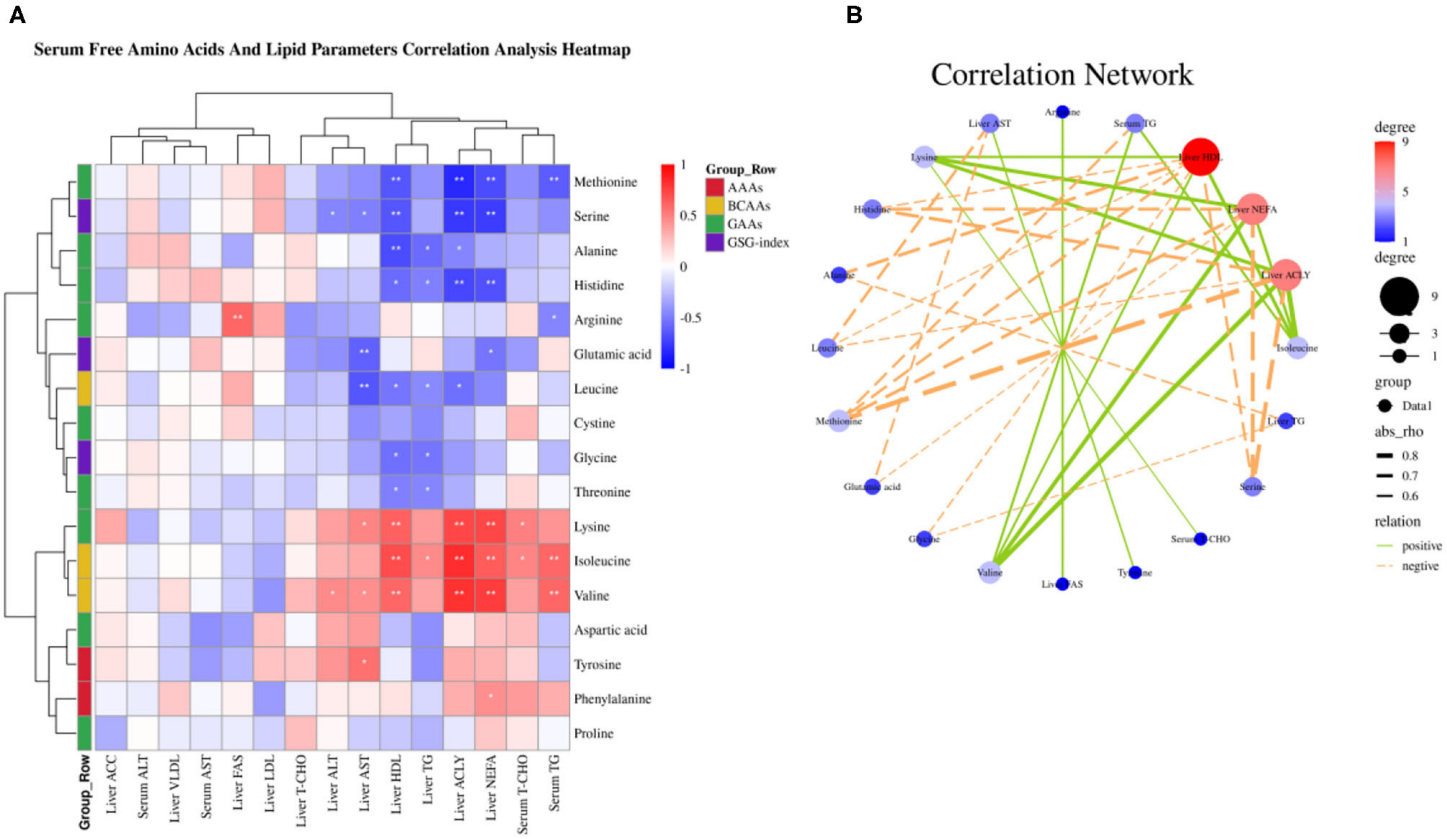
Serum-free amino acids and lipid parameters correlation analysis. **(A)** Serum-free amino acids and lipid parameters correlation analysis heatmap. **(B)** Correlation network. *P < 0.05 and **P < 0.01.

Amino acids as substrate or precursor generate acetyl-coenzyme A (CoA) *via* tricarboxylic acid (TCA) cycle participate in fatty acid metabolism, thus we sought to elaborate the relationship between serum free amino acids and fatty acid metabolism by the Spearman correlation analysis. The Spearman correlation analysis heatmap demonstrated serum free amino acids were significantly associated with serum and liver lipid metabolism parameters. As shown in Figure 4A, serum free Met was negatively associated with serum TG, liver NEFA, ACLY, and HDL (*P* < 0.01); Ser was negatively associated with liver NEFA, ACLY, HDL, ALT, and AST (*P* < 0.05); Ala was negatively associated with liver ACLY, TG, and HDL (*P* < 0.05); His was negatively associated with liver NEFA, ACLY, TG, and HDL (*P* < 0.05); Arg was negatively associated with serum TG, whereas was positively associated with liver FASN (*P* < 0.05); Glu was negatively associated with liver NEFA and AST (*P* < 0.05); Leu was negatively associated with liver ACLY, TG, HDL, and AST (*P* < 0.05); Gly was negatively associated with liver TG and HDL (*P* < 0.05); Thr was negatively associated with liver TG and HDL (*P* < 0.05); Yet, Lys was positively associated with serum T-CHO, liver NEFA, ACLY, HDL, and AST (*P* < 0.05); Ile was positively associated with serum TG and T-CHO, liver NEFA, ACLY, TG, and HDL (*P* < 0.05); Val was positively associated with serum TG, liver NEFA, ACLY, HDL, ALT, and AST (*P* < 0.05); Tyr was positively associated with liver AST (*P* < 0.05); Phe was positively associated with liver NEFA (*P* < 0.05). The correlation network analysis further revealed serum free amino acids dramatically influenced liver NEFA, ACLY, and HDL, serum-free Val, Ile, and Lys play a positive role, yet Met, Ser, His, Leu, and Ala plays a negative role (Figure 4B). Overall, these results suggested that circulating amino acid quantity—and in particular, the elevated concentrations of Val, Ile, and Lys in serum circulation—may be an important accelerant of fatty acid synthesis.

### Dietary Valine Promoted Lipogenesis Trigger NAFLD *via* Repressing FGF19-TORC1 Signaling Pathway Mediated by GCN2-EIF2α-ATF4

**Figure 5:**
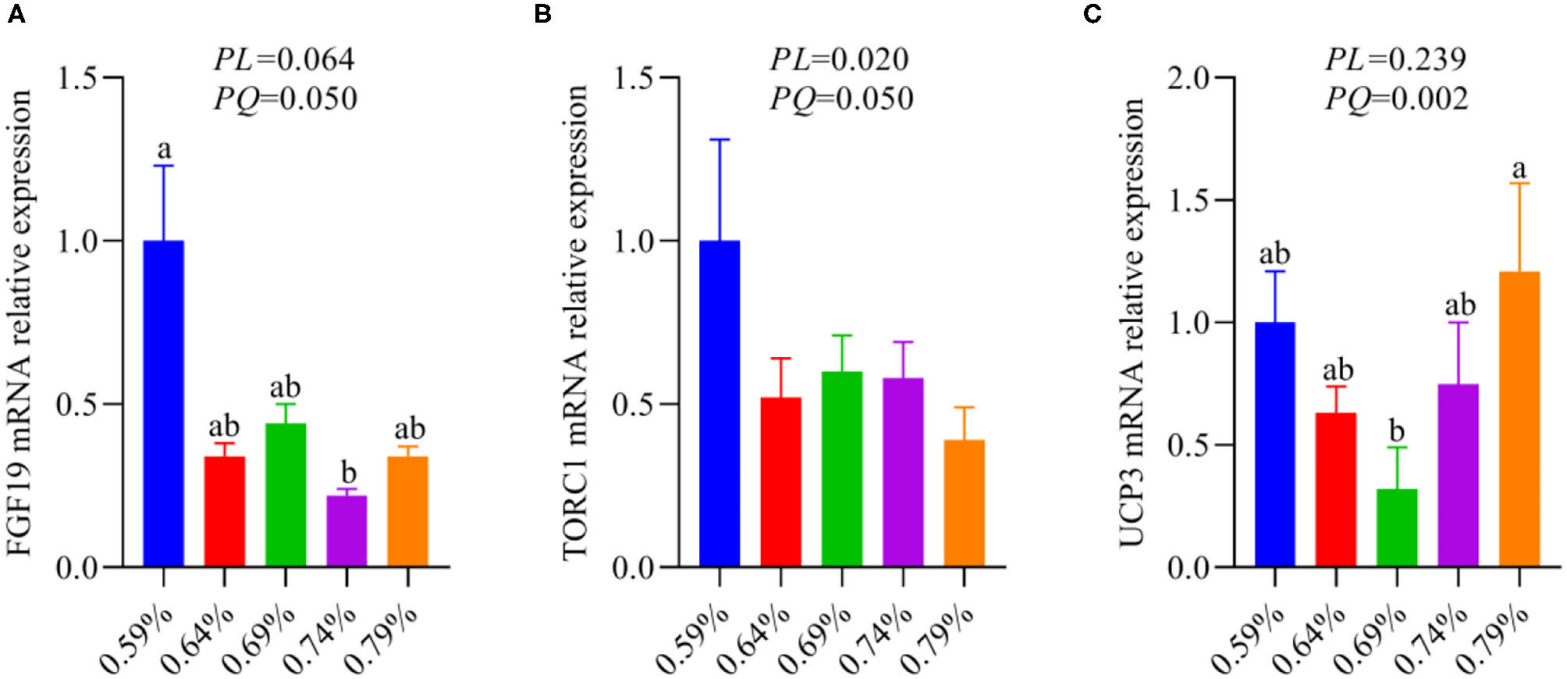
The relative mRNA expression levels of an FGF19-TORC1 signaling pathway (*n* = 6–8). **(A)** FGF19. **(B)** TORC1. **(C)** UCP3. ^a, b^Means with different superscripts within a column differ significantly (*P* < 0.05).

Our recent report indicated that low levels of dietary valine activated the GCN2-eIF2α-ATF4 pathway, while high levels of dietary valine inhibited it (33). Fibroblast growth factor family (FGF), for instance, FGF21 and FGF19 (37). FGF21 is a target gene of ATF4 and is critical to the adaptive metabolic response to amino acid deprivation (37–39). FGF19 is a late-fed-state gut hormone that is induced by the bile acid nuclear receptor which can inhibit lipogenesis (39). GCN2 has also been connected to mTORC1 activation and activation of the GCN2-ATF4 signaling pathway in amino acid-deprived cells results in mTORC1 inhibition (40). In addition, FGF21 can repress insulin- or nutrient-stimulated activation of mTORC1 in the liver (41). Last research suggested that each BCAA has distinct metabolic effects, and a low isoleucine diet reprograms liver and adipose metabolism *via* activating the FGF21-uncoupling protein 1 (UCP1) axis (42). Reducing valine induces similar but more modest metabolic effects, whereas these effects are absent with low leucine (42). Therefore, we further investigated whether dietary valine influences the FGF19-TORC1 or UCP3 axis and whether it is mediated by the GCN2-eIF2α-ATF4 pathway, while the FGF21 and UCP1 are not detected due to the chicken genome lack of them. With increasing dietary valine levels, our RT-PCR revealed that dietary valine supplementation dramatically downregulated the mRNA expression levels of FGF19 and TORC1 (Figures 5A,B), yet the UCP3 showed first downregulated and then upregulated in a quadratic manner (*P* < 0.05) (Figure 5C). Taken together with our previous results, we found that excessive dietary valine stimulated lipogenesis drives NAFLD by inhibiting the FGF19-TORC1 pathway in a GCN2-eIF2α-ATF4 dependent manner.

### NAFLD Induced by Excessive Dietary Valine Repressed Autophagy *via* GCN2-FGF19-TORC1 Results in Enhanced Inflammatory Response

**Figure 6:**
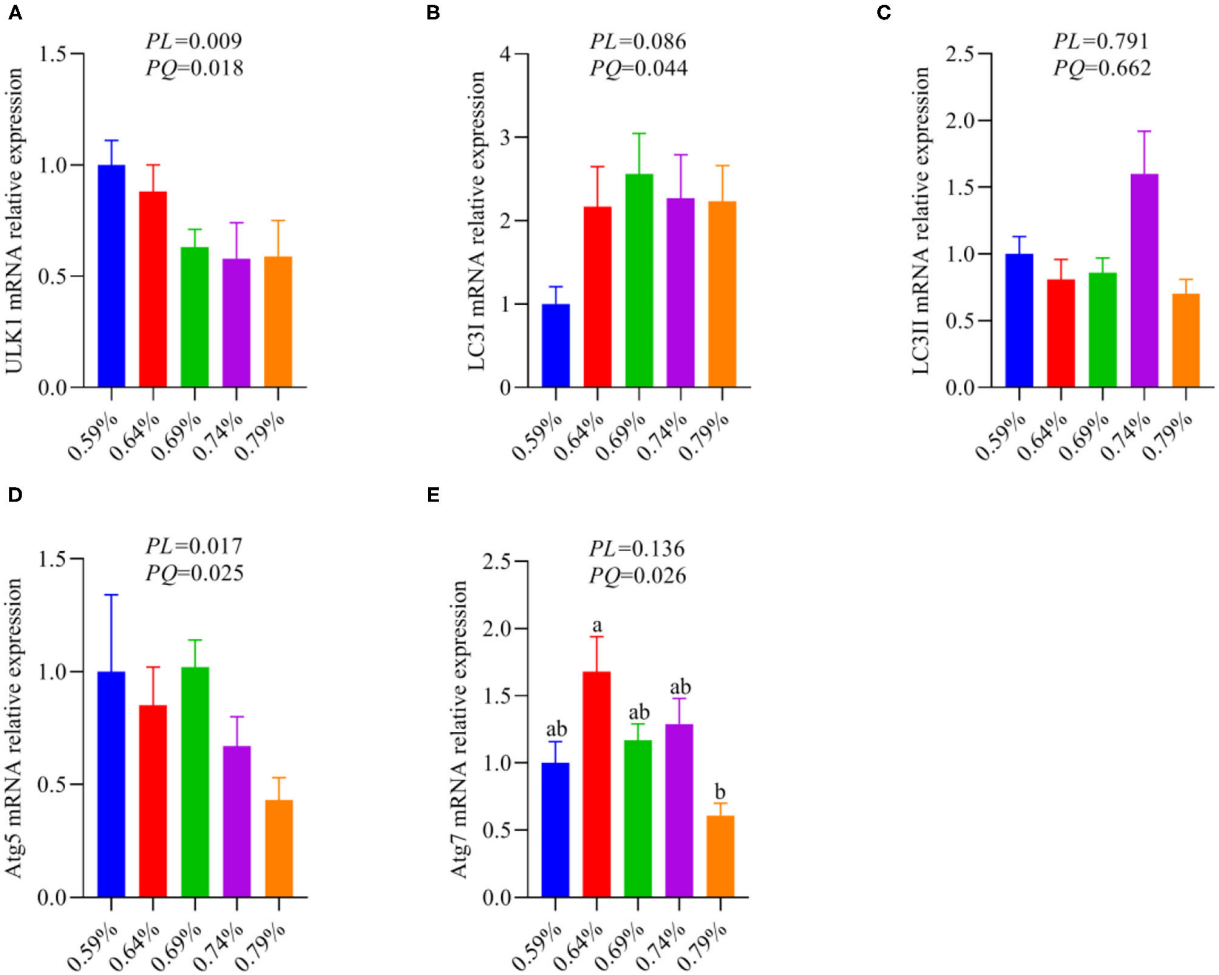
The relative mRNA expression levels of an autophagy signaling pathway (*n* = 6–8). **(A)** ULK1. **(B)** LC3I. **(C)** LC3II. **(D)** Atg5. **(E)** Atg7. ^a, b^Means with different superscripts within a column differ significantly (*P* < 0.05).

As an amino acid sensor, GCN2 is sensitive to a paucity of one or more essential amino acids to inhibit mTORC1, resulting in autophagy induction (29). The mTORC1 activation promotes phosphorylation of ULK1, an upstream regulator of autophagosome biogenesis, thereby inhibiting autophagy (11, 27). Based on the results of Figure 5, thus, we further analyze whether inhibition of the GCN2-FGF19-TORC1 signaling pathway affects autophagy. We found that dietary valine treatment significantly downregulated the mRNA expression levels of ULK1, LC3I, Atg5, and Atg7 in a linear or quadratic manner with the increase of dietary valine levels (*P* < 0.05) (Figures 6A,B,D,E), whereas did not impact LC3II mRNA expression level (*P* > 0.05) (Figure 6C). Lipotoxicity induced by excessive lipids commonly triggers inflammation reaction and eventually induces NASH (5, 6). GCN2-autophagy defect can trigger the production of pro-inflammatory cytokines such as IL-β and IL-17 (28). Our previous results confirmed that NAFLD induced by high levels of dietary valine significantly increased the liver concentrations of IL-β and IL-17 in a quadratic manner (33). Given both the inhibition of the GCN2-FGF19-TORC1-autophagy signaling pathway and enhanced liver inflammatory response, we concluded that NAFLD induced by high levels of dietary valine trigger liver inflammatory response *via* suppressing the GCN2-FGF19-TORC1-autophagy signaling pathway.

### Amino Acid Imbalance and NAFLD Synergistically Trigger Oxidative Stress

**Figure 7:**
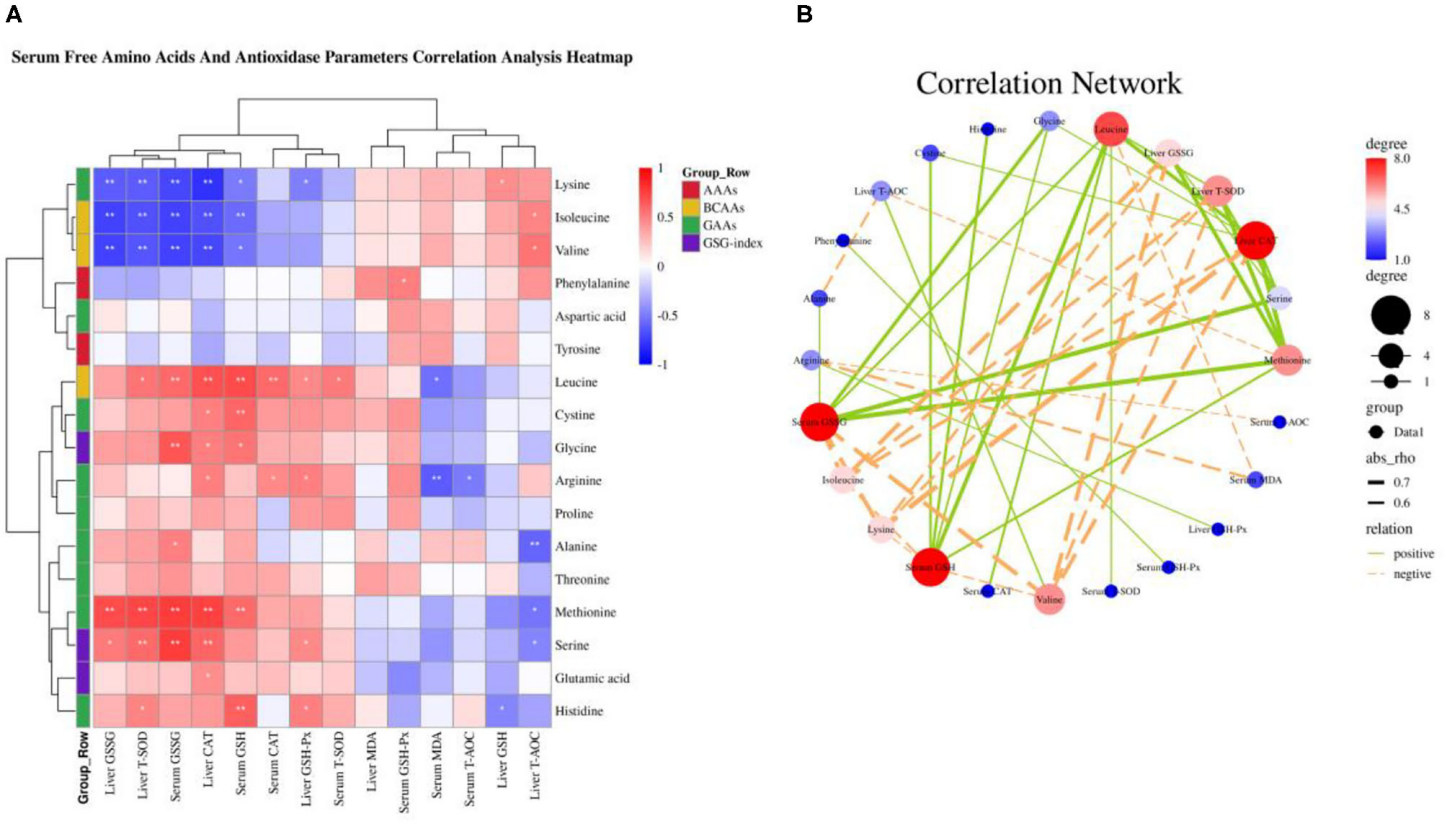
Serum-free amino acids and anti-oxidase parameters correlation analysis. **(A)** Serum-free amino acids and anti-oxidase parameters correlation analysis heatmap. **(B)** Correlation network. *P < 0.05 and **P < 0.01.

A “second hit” of oxidative stress induced by lipid toxicity leads to the generation of high levels of reactive oxygen species (ROS), which further accelerate the NAFLD transform to NASH (5, 6). Inhibition of autophagy accelerates the production of ROS and decreased amino acids including glutamine, glutamate, cysteine, glycine, threonine, and serine, which suppress the production of GSH synergistically promote the severity of oxidative stress (15, 20–23). Based on our previous research that dietary valine treatment significantly inhibited the production of anti-oxidases and the concentrations of serum and liver GSH and GSSG (32, 33). Therefore, here we attempt to analyze the relationship between changed serum free amino acid profile and body anti-oxidases by correlation analysis. As shown in Figure 7A, the Spearman correlation analysis revealed that changed serum free amino acid profile dramatically associated with altered body anti-oxidases. Correlation heatmap indicated that serum free Lys was positively associated with liver GSH, but was negatively associated with serum GSH and GSSG, and liver GSH-Px, CAT, T-SOD, and GSSG (*P* < 0.05); Ile and Val were positively associated with liver T-AOC, while were negatively associated with serum GSH and GSSG, and liver GSH-Px, CAT, T-SOD, and GSSG (*P* < 0.05); Phe was positively associated with serum GSH-Px (*P* < 0.05); Leu was positively associated with serum T-SOD, CAT, GSH, and GSSG, and liver GSH-Px and T-SOD, yet was negatively associated with serum MDA (*P* < 0.05); Cys was positively associated with serum GSH and liver CAT (*P* < 0.05); Gly was positively associated with serum GSH and GSSG, and liver CAT (*P* < 0.05); Arg was positively associated with serum CAT and liver GSH-Px and CAT, whereas was negatively associated with serum T-AOC and MDA (*P* < 0.05); Ala was positively associated with serum GSSG, while was negatively associated with liver T-AOC (*P* < 0.05); Met was positively associated with serum GSH and GSSG, and liver CAT, T-SOD, and GSSG, but was negatively associated with liver T-AOC (*P* < 0.05); Ser was positively associated with serum GSSG, and liver GSH-Px, CAT, T-SOD, and GSSG, yet was negatively associated with liver T-AOC (*P* < 0.05); Glu was positively associated with liver CAT (*P* < 0.05); His was positively associated with serum GSH, and liver GSH-Px and T-SOD, while was negatively associated with liver GSH (*P* < 0.05). Furthermore, the correlation network analysis found that serum-free amino acids significantly affected serum GSH and GSSG, and liver CAT, T-SOD, and GSSG, serum-free Leu, Met, Gly, His, Cys, and Ser plays a positive role, whereas serum-free Val, Lys, and Ile play a negative role (Figure 7B). Together with previous results, we concluded that amino acid imbalance and NAFLD synergistically accelerated oxidative stress.

## Discussion

Non-alcoholic fatty liver disease commonly occurs in humans with obesity and T2DM, as well as in animals including rodents and laying hens. This condition is often accompanied by systemic metabolic disorders, such as excessive lipids deposition, steatosis, insulin resistance, oxidative stress, inflammatory response, ER stress, and autophagy inhibition (2–4). Perturbations in amino acid metabolism were suggested to be implicated in the pathogenesis of NAFLD and progression to NASH (23, 43, 44). In particular, elevated circulating BCAAs and AAAs and lower circulating glutamine, glutamate, alanine, aspartate, and glycine have been consistently reported in patients with NAFLD (12, 16–23), whereas single BCAA such as valine how to affect the development of NAFLD has not been systematically addressed so far. Here, we integrated our previous results and further identified that changed serum amino acid profile and impaired amino acid metabolism were significantly associated with fatty acid metabolism, oxidative stress, and inflammatory response mediated by the GCN2-FGF19-TORC1-autophagy signaling pathway. Using the Spearman correlation analysis and dietary intervention approaches to change dietary valine levels, we provide evidence for a causative role of valine in NAFLD of laying hens.

Based on our previous results that dietary valine supplementation dramatically influenced serum amino acid profile (32). For instance, dietary valine treatment significantly elevated serum BCAAs including valine and isoleucine, and AAAs such as phenylalanine and tyrosine (32). It has been demonstrated that increased concentrations of BCAAs contribute to insulin resistance in obese and T2DM humans and were associated with an increased risk of NAFLD (16–18). However, Solon-Biet and colleagues found that long-term exposure to high BCAA diets leads to hyperphagia and obesity and promotes hepatosteatosis and *de novo* lipogenesis (14). These effects are not due to elevated BCAA *per se* or hepatic mammalian target of rapamycin activation but instead are due to a shift in the relative quantity of dietary BCAAs and other amino acids, in particular, tryptophan and threonine (14). Consistent with our previous results that dietary valine feed significantly decreased the serum concentration of threonine, whereas we did not determine tryptophan due to the limitation of the detection method (32). Furthermore, our Spearman correlation analysis revealed that serum-free threonine was positively associated with Asp, Gly, Ala, Cys, and Leu, which was further confirmed by decreased contents of Asp, Gly, Ala, Cys, and Leu in our recent research (32). The latest research indicated each BCAA has distinct metabolic effects in a low protein diet and the isoleucine and valine have similar metabolic effects, but the leucine does not have similar metabolic effects (42). It is interesting that we also found similar results that dietary valine treatment dramatically increased the concentrations of serum-free valine and isoleucine, whereas decreased the content of serum-free leucine (32). Our Spearman correlation analysis further demonstrated the serum-free leucine was positively associated with Gly, Thr, and Cys, yet was negatively associated with Lys, Ile, and Val. In combination with our results and the findings of other studies, these pieces of research may again indicate the unique metabolic effects of each individual BCAA, and future research should consider whether decreasing the ratio of Ile and Val and increasing the ratio of Leu will contribute to inhibiting the development of NAFLD. The AAAs including tryptophan, phenylalanine, and tyrosine, were found to increase with increased severity of liver diseases (19). Elevated circulating levels of AAAs contributed to promoting hepatic and adipose tissue lipids deposition and eventually accelerate the development and progress of NAFLD (4). In addition, AAAs can be degraded by the gut microbiota into phenylacetic acid (PAA), which can increase BCAA utilization and enhance hepatic lipid accumulation (45). Similarly, our previous results also revealed that dietary valine supplementation significantly increased the levels of serum-free phenylalanine and tyrosine (32), which is confirmed by Spearman correlation analysis that indicated serum-free phenylalanine was positively correlated with serum-free valine, isoleucine, and cysteine, but was negatively correlated with serum-free methionine.

Apart from increased circulating levels of BCAAs and AAAs, we also reported that serum-free arginine, histidine, methionine, threonine, alanine, aspartic acid, glutamic acid, glycine, and serine showed significantly decreased upon dietary valine treatment (32). Many pieces of research have indicated that glutamine, glutamate, alanine, and aspartate are positively associated with increased hepatic insulin resistance, whereas decreased glycine and serine were found in NAFLD humans (20–23), which is partly consistent with our results. Lower circulating glycine in the patients with NAFLD was associated with impaired glycine metabolism primarily mediated by alanine-glyoxylate aminotransferase 1 (AGXT1), glycine metabolism disorder plays a causative role in the development of NAFLD (15). As a new marker of severity of liver disease independent of body mass index, glutamate–serine–glycine (GSG) index [glutamate/(serine1glycine)] is reported to correlate with hepatic insulin resistance (12). Our results suggested that dietary valine supplementation elevated serum GSG-index and showed a quadratic decrease with increasing dietary levels, which may be revealed as enhanced hepatic insulin resistance in NAFLD induced by high levels of dietary valine. The correlation network analysis revealed that dietary valine supplementation dramatically influenced the components of serum-free amino acids such as valine, isoleucine, methionine, glycine, alanine, serine, threonine, histidine, and lysine. The above mentioned results suggested that dietary valine treatment significantly altered serum-free amino acid profile and led to an amino acid imbalance. Amino acid composition imbalance may imply damage to amino acid metabolism which has been confirmed in a previous study (15). Our RT-PCR results demonstrated dietary valine treatment dramatically downregulated amino acid biosynthetic genes mRNA relative expression levels, e.g., C/EBP-β and Asns. C/EBP-β can be activated by induced translationally ATF4 (46), which has been found downregulated by high levels of dietary valine in our previous report (33). As an amino acid metabolism and transport gene, asparagine synthetase (Asns) mRNA expression is dependent upon both GCN2 and ATF4 (47), which is further confirmed by our current and previous results (33). In combination with changed serum free amino acid profile and impaired amino acid metabolism, we can conclude that dietary valine supplementation resulted in amino acid imbalance and damaged amino acid metabolism mediated by GCN2-eIF2α-ATF4.

The first step of lipid deposition is synthesizing fatty acids *via* stimulating liver FASN, ACLY, and ACC secretions, and then, the fatty acids are esterified to generate TGs, eventually to store in lipid droplets (36). We found that dietary valine supplementation dramatically elevated the contents of liver ACLY, whereas the content of liver FASN first increased and then decreased with the increase of dietary valine levels. However, dietary valine treatment did not affect the contents of liver ACC. Consistently, our previous study confirmed this result, wherein dietary valine significantly upregulated the mRNA relative expression levels of FASN and ACLY yet did not affect the mRNA expression level of ACC (33). Our data of elevated NEFA further proved that dietary valine promoted fatty acid synthesis by stimulating the secretion of FASN and ACLY. Previous results have demonstrated that excess BCAAs directly contributed to *de novo* lipogenesis or *via* increased ACLY phosphorylation mediated by the branched-chain ketoacid dehydrogenase kinase (BCKDK) (48, 49). The decreased concentration and downregulated mRNA expression level of FASN at the 0.74 or 0.79% level may be due to excessive fatty acid secretion feedback inhibiting it or resulting from the downregulation of GCN2-eIF2α-ATF4 mRNA expression levels (33). ACC carboxylates acetyl-CoA to form malonyl-CoA, dietary valine did not affect the concentration and mRNA expression level of ACC may be contributing to the metabolism of valine mainly produce propionyl-CoA (18). The elevation of fatty acid contributes to *de novo* lipogenesis by synthesizing TG, which has been certificated by significantly increased serum and liver TG (33). Under conditions of excess nutrients, liver fatty acid can be esterified to produce TG, which can then be secreted as VLDL-TG or stored within lipid droplets (50). Consistently, we found dietary valine treatment effectively elevated the content of VLDL but did not affect LDL. The net retention of intrahepatic TG is a prerequisite for the development of NAFLD, which encompasses a spectrum of diseases, starting with simple steatosis, through to the development of cirrhosis and hepatocellular carcinoma (51). Patients with T2DM and those with NAFLD have been reported to have an overproduction of VLDL particles (52). Interestingly, we found dietary valine also elevated the content of HDL, although the exact mechanism is not fully understood.

In addition to the increased synthesis of fatty acids, lipids can be brought into cells *via* passive diffusion and fatty acid transport or translocase proteins, including FATPs and CD36 (53). CD36 is a transporter that plays an important role in facilitating intracellular FFA uptake and trafficking, which dysfunction has been implicated in lipophagy reduction and NAFLD development (54). As the most common fatty acid transport protein in the liver, FATP1 can function both to promote fatty acid uptake and to facilitate the import of long-chain fatty acids (LCFAs) (53). However, we found dietary valine did not affect the mRNA expression levels of FATP1 and CD36, which revealed that lipids accumulation induced by excessive dietary valine has not resulted in enhanced fatty acid uptake. We found dietary valine treatment significantly upregulated FABP1 mRNA expression level, which is a liver-specific FABP and mainly expressed in intracellular and its upregulation contributes to lipid droplets formation (55). In addition, we found dietary valine dramatically downregulated the mRNA expression levels of apolipoprotein A1 and B, which plays vital roles in lipids transport out of the liver and the utilization of lipids (56). Combining upregulated FABP1 and downregulated apoA1 and apoB, our current results may imply that dietary valine promotes lipid accumulation *via* accelerating lipid droplets formation and inhibiting lipids utilization. Our previous report suggested that dietary valine significantly inhibited fatty acids β-oxidation by downregulating the mRNA expression levels of fatty acid oxidation-related genes such as CPT1, ACOX1, and PPARα (33). Taken together with our previous and current results, we can conclude that dietary valine accelerated the development of NAFLD by promoting lipogenesis (including fatty acids, TG, and VLDL), inhibiting lipids export and utilization, and fatty acid oxidation mediated by GCN2-eIF2α-ATF4.

The role of amino acids in lipid metabolism has been well-demonstrated and understood, for instance, BCAAs, AAAs, and glycine (14, 15, 33, 42, 47–49). It has been clarified that amino acid imbalance and impaired amino acid metabolism contribute to lipid accumulation and the occurrence and development of NAFLD (14, 15). Thus, we sought to elaborate on the relationship between imbalanced amino acids and fatty acid metabolism by Spearman correlation analysis. Our correlation analysis revealed imbalanced amino acids are dramatically correlated with fatty acid metabolism. Serum-free valine, isoleucine, and lysine, in particular, were positively associated with liver NEFA, ACLY, and HDL, whereas serum-free methionine, serine, and histidine were negatively associated with liver NEFA, ACLY, and HDL. The correlation network further confirmed dietary valine supplementation mainly contributes to elevating liver HDL, NEFA, and ACLY mediated by elevating valine, isoleucine, and lysine levels. Long-term exposure to excessive valine diets led to amino acid imbalance, impaired amino acid metabolism, and enhanced NAFLD, which were maybe induced by a shift in the relative quantity of dietary valine, isoleucine, and lysine and other amino acids, notably methionine, serine, and histidine. For example, imbalanced amino acids drive hyperphagia and shorten lifespan due to a shift in the relative quantity of dietary BCAAs and tryptophan and threonine (14). However, there are no more reports about dietary lysine, methionine, serine, and histidine how to influence lipid metabolism. Further research will be required to fully define the mechanisms by which dietary lysine, methionine, serine, and histidine regulate the amino acid and lipid metabolism, and whether is mediated by GCN2-eIF2α-ATF4.

As a target gene of ATF4, FGF21 is critical to the adaptive metabolic response to amino acid deprivation, while the chicken genome lacks FGF21 (37). FGF19 is a late-fed-state gut hormone that is induced by the bile acid nuclear receptor which can inhibit lipogenesis (37). Our results suggested that dietary valine significantly downregulated liver FGF19 mRNA expression level, which is consistent with a previous report (39). The report found that GCN2 can connect to mTORC1 activation and activation of the GCN2-ATF4 signaling pathway in amino acid-deprived cells results in mTORC1 inhibition (40). In addition, FGF21 can repress insulin-or nutrient-stimulated activation of mTORC1 in the liver (41). Consistent with the mRNA expression level of FGF19, we found dietary valine also dramatically downregulated liver TORC1 expression level. Together with our previous results that high levels of dietary valine significantly inhibited GCN2-eIF2α-ATF4 expression levels, which may indicate the FGF19-TORC1 is the downstream target genes of GCN2-eIF2α-ATF4. Recent research suggested that a low isoleucine diet reprograms liver and adipose metabolism by increasing hepatic insulin sensitivity and ketogenesis, increasing energy expenditure, and activating the FGF21-UCP1 axis (42). Reducing valine induces similar but more modest metabolic effects, whereas these effects are absent with low leucine (42). Herein, we found the mRNA expression level of UCP3 is in contrast to GCN2-eIF2α-ATF4, which were maybe induced by the inhibition of GCN2-eIF2α-ATF4, and eventually result in the activation of UCP3.

General control non-derepressible 2 is a classic amino acid sensor and is sensitive to a paucity of one or more essential amino acids to inhibit mTORC1, resulting in autophagy induction (29). The activation of mTORC1 promotes phosphorylation of ULK1, an upstream regulator of autophagosome biogenesis, thereby inhibiting autophagy (11, 27). Based on inhibited expression levels of GCN2-eIF2α-ATF4-FGF19-TORC1, we further analyze the effects of dietary valine levels on the autophagy signaling pathway. We found dietary valine significantly downregulated the mRNA expression levels of ULK1, LC3I, and autophagy-associated proteins such as Atg5 and Atg7 yet did not impact the LC3II expression level. In NAFLD, the excess of triglycerides and FFAs suppresses the initiation of autophagy through activation of mammalian target of rapamycin (mTOR) and the suppression of serine/threonine-protein kinase ULK1 activity, leading to increased hepatic oxidative stress (57, 58). Consistent with our results that dietary valine significantly downregulated the mRNA expression levels of TORC1 and ULK1. Mice with a hepatocyte-specific knockout of Atg7 or cultured mouse hepatocytes with knock-down of Atg7 or Atg5 expression have revealed that inhibition of autophagy increases triglyceride storage in lipid droplets (59). Herein, we found dietary valine treatment dramatically inhibited the mRNA expression levels of Atg5 and Atg7, which may be induced by the inhibition of GCN2-eIF2α-ATF4-FGF19-TORC1. It has been demonstrated that LC3 was localized on the surface of LDs and the LC3 conjugation system is essential for lipid metabolism mediated by macroautophagy *via* LD formation (60). We found dietary valine significantly downregulated the mRNA expression level of LC3I, whereas did not affect LC3II. The mTORC1-ULK1 plays a vital role in autophagy by regulating autophagolyosome formation. LC3 is cleaved by ATG4 to form LC3I, and LC3I then conjugates with phosphatidylethanolamine *via* ATG7 and ATG3 to form LC3II (61). The ATG12 complex promotes LC3II formation and its conjugation to the phagosome (61, 62). NAFLD induced by excessive dietary valine did not affect the expression level of LC3II may be associated with the formation of the ATG12 complex. Future research should consider the effect of the excessive individual BCAAs on the autophagic flux and the formation of ATG12 complex *in vitro* hepatocytes. Inhibition of autophagy trigger hepatic inflammation and liver injury, which has been our previous report proved that NAFLD induced by excessive dietary valine showed enhanced inflammatory response mediated by the production of IL-1β and IL-17 *via* inhibiting GCN2 (33). In the presence of NASH, inflammation and oxidative stress are increased. Defects in macrophage autophagy induced by Atg5 knockout promoted hepatic inflammation and pro-inflammatory M1 macrophage polarization and decreased anti-inflammatory M2 macrophage polarization, resulting in the onset of liver injury in mice (63). In combination with autophagy inhibition and enhanced inflammatory response, we conclude that NAFLD induced by excessive dietary valine accelerated liver inflammation by inhibiting GCN2-TORC1-autophagy signaling pathways.

In addition to enhanced inflammatory response, lipids excess also inhibits autophagy mediated by mTORC1 and autophagy-associated proteins, resulting in increased hepatic oxidative stress (57, 58), which has been our and others reports confirmed (33, 57, 58). Our report suggested dietary valine treatment accelerated oxidative stress induced by NAFLD *via* inhibiting anti-oxidase production (33). Apart from increased hepatic oxidative stress induced by autophagy inhibition, amino acid imbalance and impaired amino acid metabolism also promote oxidative stress by increasing ROS levels. As an anti-oxidant molecule, GSH is produced by several tissues in response to oxidative stress and increased production of ROS (64). ROS stimulates the synthesis of GSH from cysteine, glycine, and glutamate, which are produced from the transamination of alanine and aspartate (12). Our previous report indicated dietary valine treatment significantly decreased serum free cysteine, glycine, glutamate, alanine, and aspartate, which is consistent with recent reports (12). Here, we revealed serum increased free valine, isoleucine, and lysine are dramatic negatively associated with decreased serum GSH and GSSG, and liver CAT, T-SOD, and GSSG by Spearman correlation analysis. In contrast, serum decreased free leucine, cysteine, glycine, arginine, methionine, serine, and histidine were significantly positively correlated with decreased serum and liver anti-oxidases. Studies have shown that metabolic dysregulation is often associated with a reduction in glycine and serine concentrations and an increase in the levels of valine and leucine (23, 65, 66), which is similar to our results (33). A recent cohort of subjects with NAFLD but without diabetes found reduced concentrations of glycine and serine (a precursor of cysteine) (12). In addition, as the limiting step for GSH synthesis (22), Glycine also plays an important role in metabolic regulation and intracellular redox balance (64), and decreased glycine has been found in NAFLD subjects (15). Correlation network analysis demonstrated dietary valine supplementation mainly contributes to promoting the production of anti-oxidases such as GSH, GSSG, CAT, and T-SOD by decreasing leucine, cysteine, glycine, arginine, methionine, serine, and histidine, whereas valine, isoleucine, and lysine suppress it. Oxidative stress usually results in liver damage and is characterized by mitochondrial dysfunction, impaired oxidation, and the production of ROS (65, 66). This process increases the demand for GSH synthesis to counteract the production of ROS by increasing intracellular GSH turnover (64). Amino acids, including glycine, serine, and glutamate, are used for the synthesis of GSH. Our results showed decreased serum free glycine, serine, and glutamate is consistent with decreased GSH and GSSG in the serum and liver (32, 33). An additional question is that decreased serum leucine was distinct from increased isoleucine and valine, which has been demonstrated in recent research that leucine has distinct metabolic effects from isoleucine and valine (42). In addition, we also surprisingly found serum lysine is dramatically increased and was positively associated with isoleucine, valine, liver NEFA, ACLY, HDL, and AST, whereas was negatively correlated with serum GSH and GSSG, and liver CAT, T-SOD, GSSG, and GSH-Px. Future research should focus on the effects of the individual BCAAs on the occurrence and development of NAFLD and its adverse metabolic impact whether is mediated by lysine.

**Figure 8:**
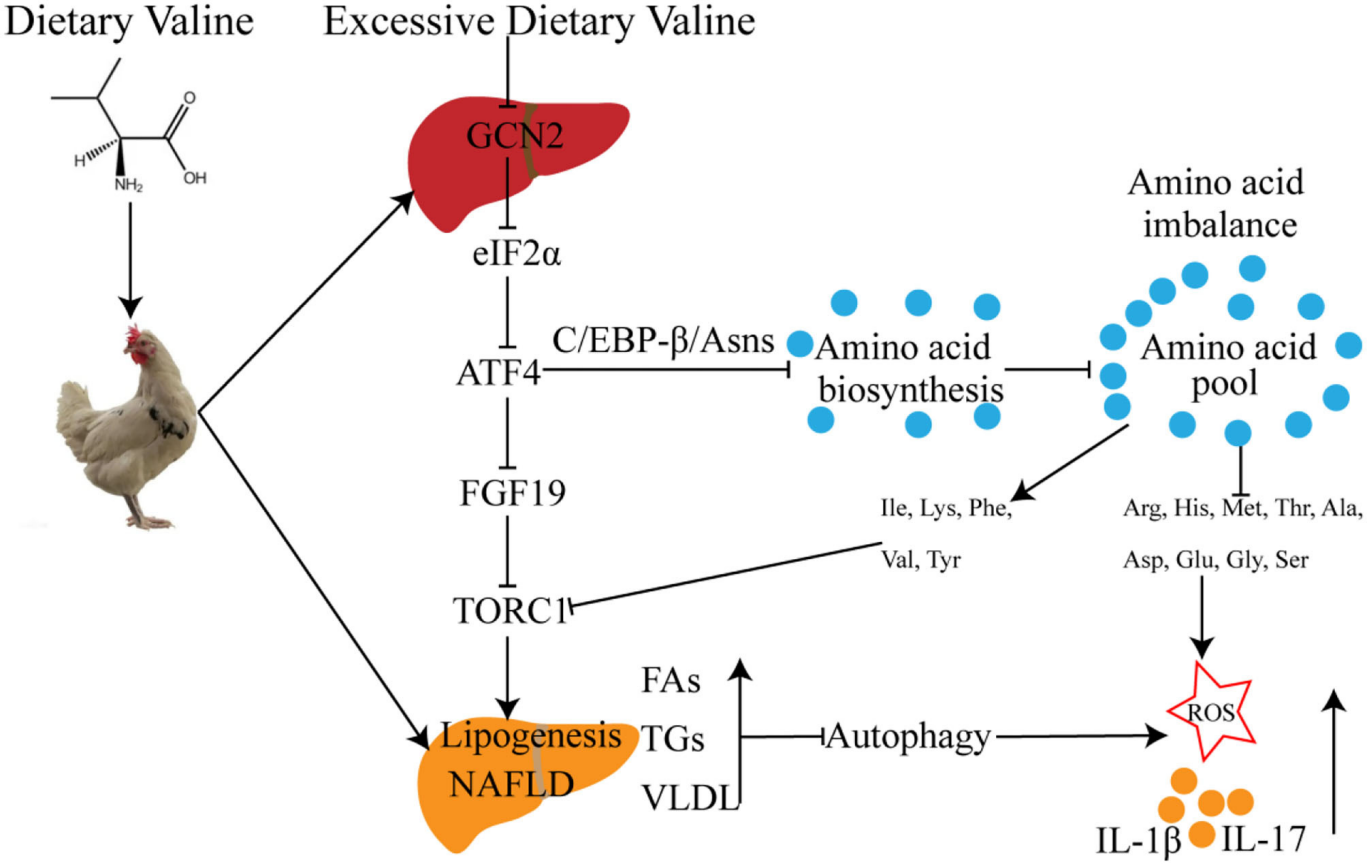
The possible molecular mechanism of dietary valine regulates amino acid and fatty acid metabolism mediated by the GCN2-TORC1-Autophagy pathway in NAFLD of laying hens.

In summary, we further demonstrated that long-term exposure to excessive dietary valine influenced amino acid and fatty acid metabolism in laying hens by inducing amino acid imbalance, impairing amino acid metabolism, promoting fatty acid synthesis, and inhibiting fatty acid utilization mediated by GCN2-FGF19-TORC1 signaling pathways. These results further lead to the occurrence and development of NAFLD, which triggers oxidative stress and inflammatory response *via* repressing autophagy pathway (Figure 8). Our results revealed the adverse metabolic effects of excessive dietary valine in laying hens, highlighting the critical importance of dietary levels of valine for the adverse metabolic function of NAFLD, which is due to the inhibition of GCN2-TORC1-autophagy signaling pathways. Finally, we suggest that reducing dietary valine may be a novel preventive and therapeutic strategy to combat the twin epidemics of NAFLD and FLHS in laying hens.

## Data Availability Statement

The original contributions presented in the study are included in the article/supplementary material, further inquiries can be directed to the corresponding author.

## Ethics Statement

The animal study was reviewed and approved by No. ZJU2013105002.

## Author Contributions

HJ: conceptualization, data curation, investigation, writing the original draft, reviewing, and editing. QX: data curation, methodology, writing, reviewing, and editing. XW: conceptualization, data curation, and methodology. YLiu: data curation and investigation. SM: methodology and investigation. YLi: data curation. TM: investigation. XD: investigation and writing, reviewing, and editing. XZ: project administration, conceptualization, funding acquisition, writing, reviewing, and editing. All authors have read and agreed to the published version of the manuscript.

## Funding

This study was supported by the Earmarked Fund for Modern Agro-Industry Technology Research System of China (CARS-40-K10).

## Conflict of Interest

The authors declare that the research was conducted in the absence of any commercial or financial relationships that could be construed as a potential conflict of interest.

## Publisher's Note

All claims expressed in this article are solely those of the authors and do not necessarily represent those of their affiliated organizations, or those of the publisher, the editors and the reviewers. Any product that may be evaluated in this article, or claim that may be made by its manufacturer, is not guaranteed or endorsed by the publisher.
